# Hahnemann Not the Founder of Isopathy

**Published:** 1883-12

**Authors:** 


To elucidate these words of Hahnemann, we quote them in full: "In the subsequent list of anti-psoric remedies, no *isopathic* remedies are mentioned, for the reason that their effects on the healthy organism have not been sufficiently ascertained. Even the itch miasm (psorin), in its various degrees of potency, comes under this objection. I call psorin a *homœopathic* anti-psoric, because if the preparations of psorin did not alter its nature to that of a homœopathic remedy, it never could have any effect upon an organism tainted with that same, identical virus. The psoric virus, by undergoing the processes of trituration and shaking, becomes just as much altered in its nature as gold does, the homœpathic preparations of which are not inert substances in the animal economy, but powerfully acting agents.

"Psorin is a *simillimum* of the itch virus. There is no intermediate degree between *idem* and *simillimum:* in other words, the thinking man sees that simillimum is the medium between simile and idem. The only definite meaning which the terms 'isopathic and æquale' can convey, is that of simillimum. They are not '*idem.*'"

In the above paragraphs, Hahnemann makes three distinct declarations:

1. He will not even mention certain remedies *because "their effects on the healthy organisms have not been sufficiently ascertained.*" Can a stronger condemnation of the use of unproved remedies be made?

2. Potentization makes inert agents active; to prove this, he cites the example of gold. Does Hahnemann claim that potentization renders gold homœopathic to any *disease?* Never. He merely asserts that trituration causes it to be a "powerfully active agent."

3. "Psorin is the simillimum of the itch virus." That is, being proved, and being prescribed upon this proving, and being made "active" by potentization, it then becomes homœopathic to the case.

